# SARS-CoV-2 Genomic Epidemiology Dashboards: A Review of Functionality and Technological Frameworks for the Public Health Response

**DOI:** 10.3390/genes15070876

**Published:** 2024-07-03

**Authors:** Nikita Sitharam, Houriiyah Tegally, Danilo de Castro Silva, Cheryl Baxter, Tulio de Oliveira, Joicymara S. Xavier

**Affiliations:** 1 Centre for Epidemic Response and Innovation (CERI), School for Data Science and Computational Thinking, Stellenbosch University, Stellenbosch 7600, South Africa; nikitasitharam1@gmail.com (N.S.);; 2 Department of Computer Science, Faculty of Science, Stellenbosch University, Stellenbosch 7600, South Africa; 3 Centre for the AIDS Programme of Research in South Africa (CAPRISA), Durban 4001, South Africa; 4 KwaZulu-Natal Research Innovation and Sequencing Platform (KRISP), Nelson R Mandela School of Medicine, University of KwaZulu-Natal, Durban 4001, South Africa; 5 Department of Global Health, University of Washington, Seattle, WA 98105, USA; 6 Institute of Agricultural Sciences, Universidade Federal dos Vales do Jequitinhonha e Mucuri (UFVJM), Unaí 38610-000, Brazil; 7 Institute of Biological Sciences, Universidade Federal de Minas Gerais (UFMG), Belo Horizonte 31270-901, Brazil

**Keywords:** SARS-CoV-2, public health informatics, computational dashboards, genomics, epidemiology

## Abstract

During the coronavirus disease 2019 (COVID-19) pandemic, the number and types of dashboards produced increased to convey complex information using digestible visualizations. The pandemic saw a notable increase in genomic surveillance data, which genomic epidemiology dashboards presented in an easily interpretable manner. These dashboards have the potential to increase the transparency between the scientists producing pathogen genomic data and policymakers, public health stakeholders, and the public. This scoping review discusses the data presented, functional and visual features, and the computational architecture of six publicly available SARS-CoV-2 genomic epidemiology dashboards. We found three main types of genomic epidemiology dashboards: phylogenetic, genomic surveillance, and mutational. We found that data were sourced from different databases, such as GISAID, GenBank, and specific country databases, and these dashboards were produced for specific geographic locations. The key performance indicators and visualization used were specific to the type of genomic epidemiology dashboard. The computational architecture of the dashboards was created according to the needs of the end user. The genomic surveillance of pathogens is set to become a more common tool used to track ongoing and future outbreaks, and genomic epidemiology dashboards are powerful and adaptable resources that can be used in the public health response.

## 1. Introduction

Infectious disease surveillance and public health communication play an important role in the prevention and control of epidemics [1,2]. Disease surveillance data must be shared promptly and in an easily interpretable way to affect changes in the public health response [3]. Public Health Informatics (PHI) is an emerging field that plays a major role in ensuring the timely dissemination of information, health promotion, and infectious disease prevention. This is achieved through the integration of information science, technology, and engineering [3,4,5]. The broad scope of this emerging field covers data translation and communication infrastructure, from conceptualization to refinement and maintenance [4,5,6]. An important tool utilized for communication in PHI is the computational dashboard [7]. Dashboards can be described as a dynamic mode, in which information and data are presented visually [8]. They provide the most pertinent information in a concise manner using data mining technologies, and this allows for an increase in decision-making power among stakeholders and policymakers [9]. In epidemiological surveillance, the usefulness of a dashboard can be seen in the accountability and transparency created between researchers, public health stakeholders, and the public [7].

Coronavirus disease 2019 (COVID-19) was caused by the virus SARS-CoV-2 [10,11,12]. This virus emerged in December 2019 in the city of Wuhan, China, and began as a clustered outbreak of viral pneumonia in a live animal market in the city [13]. By 1 March 2024, over 774 million global COVID-19 cases and over seven million COVID-19-associated deaths had been reported [14]. Genomic surveillance played an important role in understanding the COVID-19 pandemic [11,15,16]. The real-time sharing and access to genomic data during the COVID-19 pandemic enabled global monitoring of the virus’s spread and evolution [15].

During the pandemic, there was a significant increase in the number of sequences produced [15], with 57,845 sequences being deposited to the Global Initiative on Sharing Avian Influenza Data database (GISAID) in the first six months of 2020. By the end of the first year, there was a 20% increase in genomic sequences, with 314,310 sequences deposited. As of 1 March 2024, there are just over 16.5 million sequences deposited on GISAID [17]. Global genomic surveillance data, combined with epidemiological data of the SARS-CoV-2 virus, allowed for patterns of viral transmission to be elucidated and for Variants of Concern (VOCs) to be identified and tracked. For example, South Africa played a leading role in identifying VOCs and variants of interest (VOIs), which informed public health policies (such as what vaccines to administer) [11,16,18].

In the past, genomic surveillance data were not released to the public immediately. Following the outbreaks of the Ebola and Zika viruses (in 2013 and 2015, respectively), the importance and advantage of open and immediate sharing of genomic data became apparent [19]. Sharing genomic epidemiological data publicly, in a timely fashion, is particularly useful for reconstructing precise epidemic transmission dynamics and subsequently allowing informed decision-making about interventions and control measures [20,21]. However, it can be difficult for the public and professionals, who do not specialize in this field, to interpret these data [22]. Therefore, presenting this information in an easily interpretable manner on a dashboard would be effective for situational awareness, public health communication, and influencing policy [22,23]. During the SARS-CoV-2 pandemic, genomic epidemiology dashboards were used in data-driven public health decisions.

This review aims to provide users of genomic epidemiology dashboards with an understanding of the functionality and technologies of public health dashboards, in the context of publicly accessible SARS-CoV-2 dashboards. Here, we evaluate six dashboards to provide a better understanding of the data presented, visual and functional features, and software used. We finalize this review by highlighting how dashboards can be adapted to novel, emerging, or re-emerging pathogens, such as Influenza A, dengue virus, Ebola virus, and *Vibrio cholera*.

## 2. SARS-CoV-2 Genomic Epidemiology Dashboards

There was an exponential increase in the number and types of dashboards utilized during the COVID-19 pandemic [8]. Examples of these included epidemiological dashboards which presented case, death, hospitalization, and vaccination data, such as the Johns Hopkins [13] and World Health Organization (WHO) dashboards [14]. Another important epidemiological dashboard that was used extensively during the pandemic was Our World in Data’s Coronavirus Pandemic dashboard. This dashboard enabled important comparisons of key epidemiological indicators between countries [24]. Mathematical modeling dashboards such as epiMOX, which also contained a predictive tool to understand the progression of the evolution of the epidemic, were also produced [25]. A hospital management dashboard [26] was created by the New York Presbyterian-Columbia University Irving Medical Center to assist with the rapid reorganization and planning of nephrology services during the COVID-19 pandemic. Various open-source tools were developed for this dashboard to facilitate the management of resources and the supply chain for the hospital [26]. Dashboards created during the pandemic had a significant effect on how difficult data and metrics were translated to information for the public, media, and policymakers [15].

**Figure 1:**
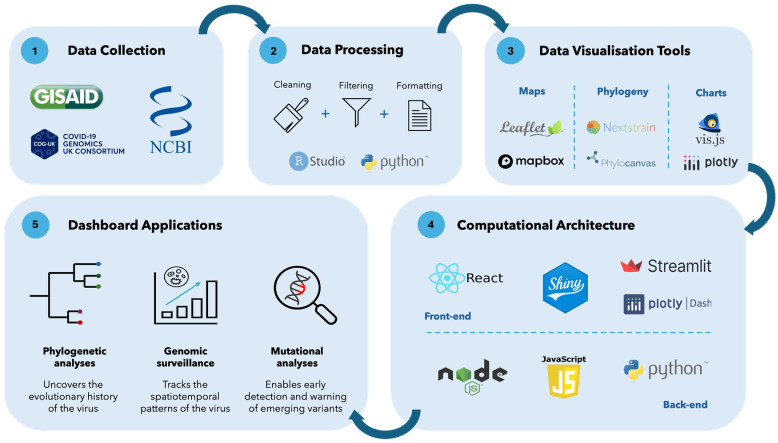
Process involved in the design and production of a genomic epidemiology dashboard. 1—Data collection, 2—processing of the source data, 3—analyzing and visualizing the data, 4—computational architecture development of the front- and back-end, and 5—different categories of genomic epidemiology dashboards.

Genomic epidemiology dashboards were also created to communicate information generated from ongoing genomic surveillance systems, given the large-scale expansion of genomic surveillance globally during the SARS-CoV-2 pandemic [21]. Figure 1 shows the general process undertaken to produce genomic epidemiology dashboards. Data (sequence data and associated metadata) are typically ingested from genomic databases. The data are then processed through three general steps: cleaning, filtering, and formatting. The processed data can then be visualized in different ways using a variety of data visualization tools. Examples of the different visualizations that are produced for genomic epidemiology dashboards include maps, phylogenetic trees, and charts. The visualizations are then integrated with the computational architecture of the dashboard. The computational architecture typically includes a front- and a back-end.

Genomic epidemiology dashboards can be broadly classified into those that display phylogenetic analyses, genomic surveillance data, and mutational data (Figure 1, Panel 5). These dashboards integrate specialized genomic analyses but present them in an interactive and easy-to-interpret way. Phylogenetic analyses are useful on a genomic epidemiology dashboard, as they depict the evolutionary history of the virus. The information produced from phylogenetic analyses is important when monitoring the genetic changes in emerging variants of a virus, which affects vaccine efficacy, rate of transmission, and viral antigenicity [27]. This information can also help characterize the levels of community transmission in a particular location or viral movements between locations. Genomic surveillance data on a dashboard are important, as they present temporal, spatial, and variant proportion and prevalence information about the genomes that are sequenced. This can provide a succinct perspective of the spread of genomic variants and can inform further surveillance and intervention efforts [28]. Mutational data of viruses are useful to present on dashboards, as they provide insight into viral virulence, infectivity, and transmissibility [29]. This is achieved through a variety of methods, such as Single Nucleotide Polymorphism (SNP) calling, relative growth advantage, and machine learning models, to predict mutations [29,30].

In this review, we selected a total of six publicly available SARS-CoV-2 dashboards that fell into the different genomic epidemiology dashboard categories. We prioritized choosing dashboards with diverse characteristics to achieve a well-rounded understanding of the utility of genomic epidemiology dashboards. The phylogenetic dashboard category included Nextstrain’s and Cluster-Tracker’s dashboards. Nextstrain’s Genomic Epidemiology of SARS-CoV-2 was selected as it displays global phylogenetic trees, nucleotide diversity, and frequencies for SARS-CoV-2. Cluster-Tracker was another diverse dashboard selected, as it displays information from a phylogenetic-informed summary heuristic for the United States of America (USA) only. The genomic surveillance dashboards included the Microreact, Wellcome Sanger Institute, and the SARS-CoV-2 Africa dashboards. MicroReact’s Global SARS-CoV-2 dashboard is a simple, one-page dashboard that displays global genomic information on the virus’s variants geographically and temporally. The Wellcome Sanger Institute’s COVID-19 Genomic Surveillance dashboard was selected as it displays genomic and estimated case information for the United Kingdom (UK) only. The SARS-CoV-2 Africa dashboard was selected as it focuses on SARS-CoV-2 genomic epidemiology for Africa only from a temporal and geographical view. A mutational dashboard included in this review is the CoV-Spectrum dashboard. CoV-Spectrum is a detailed dashboard focusing on the globally known SARS-CoV-2 variants, their amino acid and nucleotide substitutions, deletions, and insertions.

We evaluated three major properties of genomic epidemiology dashboards, with a closer look at specific elements within these properties. These properties included the data, visual and functional features, and computational architecture on the genomic epidemiology dashboards. The selected six SARS-CoV-2 dashboards were drawn on as examples when discussing the specific elements within the overarching properties, and a comprehensive summary is provided in Table 1.

## 3. Data on Genomic Epidemiology Dashboards

### 3.1. Data Sources

The quality of data determines the reliability of a dashboard. Presenting reliable data in an easy-to-interpret manner allows the dashboard to be used as a data-driven tool in policy decision-making. This would alleviate information asymmetry between multiple stakeholders [7,31].

The two most common databases used to provide sequence data and support genomic analyses on the selected genomic epidemiology dashboards are GenBank and GISAID (Table 1) [32,33]. These databases have different approaches to how data can be reshared, with principles based on ensuring open science and data sharing to restricting redistribution to protect the data submitters [34]. GenBank adopts open data principles as implemented by the International Nucleotide Sequence Database Collaboration (INSDC). INSDC advocates for open science and data sharing of published or unpublished results [35]. However, the immediate sharing of SARS-CoV-2 genomic data could potentially leave data submitters vulnerable to “scooping”, if analyses and publications are not produced timeously [34]. Thus, databases sharing genomic data can choose to restrict the distribution of the available data. For example, GISAID encourages submitters to share unpublished data, as the database prohibits users from redistributing the metadata or sequence data [34]. This enables genomic epidemiology dashboards to present unpublished data in near-real time, but they cannot be downloaded and shared.

Nextstrain’s dashboard is an example that utilizes both GenBank and GISAID data. The Nextstrain Team has taken it a step further and curated open datasets from GenBank. This allows users of these data to have a preprocessed starting point from which other analyses can be conducted [34]. CoV-Spectrum is an example of a dashboard that utilizes the Nextstrain datasets as a data source [20]. The SARS-CoV-2 Africa dashboard is an example that sources data from GISAID only [36]. Microreact and Wellcome Sanger Institute’s dashboards present data sourced from the COVID-19 Genomics UK Consortium (COG-UK) [37,38]. These data are stated to be only a subset of what is made available by COG-UK and do not represent a complete record of what is available on public databases. Cluster-Tracker is a dashboard that incorporates sequence data from GenBank, GISAID, and COG-UK [39]. The genomic epidemiology dashboards that present data from GenBank, Nextstrain, or COG-UK provide the option for downloadable metadata.

### 3.2. Data Processing

How source data are processed is another factor that affects the quality of information presented on a genomic epidemiology dashboard. It is important to ensure the source data are processed appropriately; otherwise, it can lead users to arrive at incorrect conclusions [7,40]. Panel 1 in Figure 1 provides general steps that take place during data processing, often using RStudio and the Python programming language to achieve this. It is beneficial for dashboards to describe the data processing steps taken, whether as a disclaimer on a web page or in a publication. Providing this information enables users to produce datasets of the same standard for similar analyses. For example, Nextstrain’s genomic epidemiology dashboard provides detailed documentation on the workflow and tools used to process the source data on the web page. On the other hand, Cluster-Tracker’s and the SARS-CoV-2 Africa’s dashboards detail the data processing steps in a publication [36,39].

### 3.3. Geographic Level

Dashboards can be designed for various geographic levels depending on the overall purpose of the dashboard, the users it was created for, and the level of data granularity (Table 1). For example, Wellcome Sanger Institute’s dashboard presents genomic surveillance data for each local authority in the UK [38]. This level of data granularity is useful for users, as it provides a more detailed perspective on the key performance indicators (KPIs) of the dashboard. Another example of a dashboard displaying genomic surveillance data for a different geographical level is the SARS-CoV-2 Africa dashboard. This dashboard is useful, as it provides a dynamic perspective of genomic surveillance on the African continent [36].

### 3.4. Key Performance Indicators

Dashboards present data in a specific set of metrics, known as KPIs, which allow users to utilize the information in decision-making processes [41]. In the context of genomic epidemiology dashboards, KPIs are key metrics that clearly define the outbreak or epidemic situation [42]. These include, for example, the number of genomes produced, the proportion of variants over time, and the mapping of mutations within the genome.

An important part of the dashboard design process is determining the number of KPIs to include. A study by Nijkamp and Kourtit (2022) refers to the “magical number seven” [31]. This principle is based on experimental research and explains that any decision that considers more than seven objectives will be flawed and inconsistent. Thus, genomic epidemiology dashboards that have more than seven KPIs can confuse users and prevent them from making informed decisions [43].

**Figure 2:**
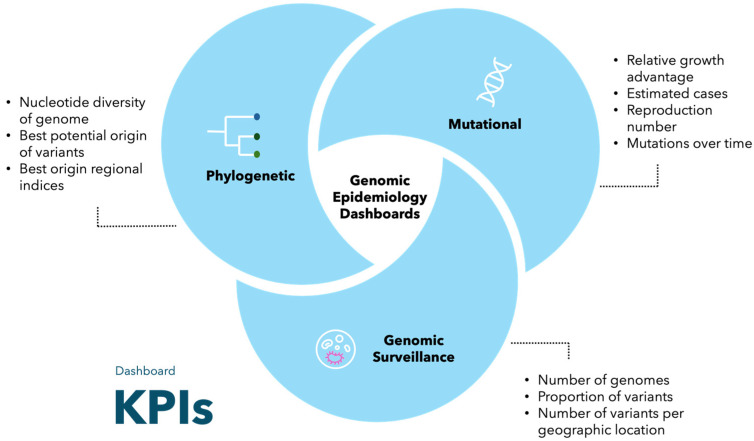
Key performance indicators (KPIs) of the different types of genomic epidemiology dashboards.

The type of KPIs presented vary over the category of genomic epidemiology dashboards, as shown in Figure 2. Genomic surveillance data dashboards typically include KPIs, such as the number of genomes produced, the proportion of variants, and the number of variants per location (region, country, state, etc.). Phylogenetic dashboards include KPIs such as the nucleotide diversity of the genome, best potential origins, and best origin regional indices. Mutational dashboards present KPIs, such as relative growth advantage, estimated cases, reproduction number, and specific information about the mutations over time. It should be noted that a genomic epidemiology dashboard can also present KPIs from across the different categories.

## 4. Visual and Functional Features

The visual features on a dashboard refer to how information is presented, and the functional features refer to how the dashboard carries out its tasks. A user needs to consider the types of analyses and filtering options of a genomic epidemiology dashboard. This will affect the user’s dashboard choice when employing it in different situations.

### 4.1. Analyses

There are a variety of analyses that can be presented within each category of genomic epidemiology dashboards. The types of analyses and visualizations are summarized in Table 1.

Phylogenetic trees are the most common visual feature found on genomic epidemiology dashboards to present information from phylogenetic analyses. Nextstain’s dashboard contains an interactive and customizable phylogenetic tree, which can be seen in Panel B in Figure S1. The subsampling for the phylogenetic tree can be changed in Panel A under the “Dataset” section. The subsampling period determines the time frame which the global sequences are selected from. This will form the focus of the phylogenetic tree with contextual sequences that accurately represent the pandemic before the subsampling period. An interactive phylogenetic tree would be useful to various users, depending on the type of information taken from it. For example, evolutionary virologists would benefit as it provides in-depth genetic history information, whereas an epidemiologist would be interested in the emerging variants to inform outbreak management [44].

Cluster-Tracker is an example of a genomic epidemiology dashboard that displays phylogenetic information without using a phylogenetic tree. Cluster-Tracker is an open-source dashboard that visualizes the results of a phylogenetically informed summary heuristic for the USA. This dashboard prioritizes communicating the results of this heuristic model, which are clusters that represent the number of introductions of the SARS-CoV-2 virus into a State; this allows for major transmission clusters of that State to be represented. Cluster-Tracker contains one visualization; an interactive map of North America is displayed, with emphasis and coloring only present in the USA (Figure S2). The map is colored according to a color ramp indicating the number of clusters. The period of this interactive visualization can be controlled by the options in the top left-hand corner. The user can extract a large amount of information for each state on the map. This includes the log-fold enrichment of introductions into the selected state, and the number of introductions from other states into the selected state (Figure S2) [39]. Although the phylogenetically informed summary heuristic is complex, the visualization allows users to track the introductions of SARS-CoV-2 into each state, without any specialized knowledge.

An example of the types of analyses a user can find on a genomic surveillance dashboard can be seen on the SARS-CoV-2 Africa dashboard. This dashboard presents information produced by SARS-CoV-2 genomic surveillance programs on the African continent. This dashboard aims to produce an interactive perspective of the SARS-CoV-2 variants circulating on the African continent [36]. Panel B in Figure S3 is a map of Africa denoting the number of genomes each country has produced, through a gradient color ramp. This map is animated and can be controlled with the play, pause, and stop buttons located beneath it. There is also a sliding time window that allows the user to toggle the date range of the data displayed. Panel C is a stacked proportional bar chart depicting the proportion of circulating lineages and variants. Panel D is a strip chart displaying the genomic sequence data available for individual African countries. This figure is a jittered scatter plot that displays the number of genomes as colored dots, where the color represents the variant of the genome; each dot represents a variable number of genomes (Figure S3) [36].

CoV-Spectrum is a dashboard that presents mutational information about the SARS-CoV-2 genome and identifies novel VOCs. This is achieved by providing a summary of the raw data and presenting subsequent statistical analyses. Figure S4 shows the three main interactive panels (B-D) that can be found on the CoV-Spectrum dashboard. Panel B, titled “Known Variants”, presents a grid of possible variants that can be explored individually. After the selection has been made, the user is redirected to another dashboard page. This page is where a user can access a vast amount of mutational data about a specific variant in a summarized format. A few examples of the data provided on each variant include the estimated cases from that variant, relative growth advantage with three different mechanisms, reproduction number, mutations over time, nucleotide entropy, insertions, substitutions, and deletions. These analyses are useful to genomic specialists looking to gain a deeper understanding of each monitored variant of SARS-CoV-2 [20].

### 4.2. Filtering Options

There is a wealth of information produced by genomic surveillance systems, and this is displayed on genomic epidemiology dashboards in an aggregated fashion. However, the dashboard must provide users with the option to further drill down into the data [40]. This enables the user to interact with the data and increases the transparency between the researcher and various stakeholders [7,31]. Advanced filtering options on genomic epidemiology dashboards ensure there are drill-down features for aggregated information that can be explored by the user. 

There are different filtering options for data across the categories of genomic epidemiology dashboards (Table 1). Filtering options on phylogenetic dashboards can include the customization of a phylogenetic tree. This can be seen on Nextstrain’s dashboard, where the user can navigate to the “Tree Options” found in Panel A (Figure S1). The customization options include changing the layout, branch length, branch labels, and tip labels of the tree. Customizing the tree can provide the user with valuable information that can be used to inform various decisions. For example, changing the tips colors to represent emerging variants can provide the user with information on which emerging variants are found in the global sample. Public health laboratory managers could use this information, for example, to request for more samples to be sequenced; this could assist in determining if the emerging variant in the global dataset correlates with the variants found in a specific country [44].

The filtering options found on genomic surveillance dashboards typically involve temporal, geographical, and variant filter options. For example, the Wellcome Sanger Institute’s dashboard contains three main filters; these can be seen in Panel A–C (Figure S5). The first filter (A) controls the timescale of the data that are presented. The second filter (B) allows the user to select a local authority or postcode from England. The third filter (C) allows for the lineages of SARS-CoV-2 to be selected. There is a lineage option map that appears in Panel D of the dashboard, once the “Lineages” settings button, in Panel C, is selected (Figure S5). This option is useful, as it enables users to drill down into temporal, geographical, and estimated case data for a specific variant. 

Mutation dashboards also contain filtering options to enable users to interact with and subset the data presented on the dashboard. Figure S6 shows the advanced filtering option page of CoV-Spectrum. These advanced filters deal with hosts, submission dates of sequences, and various sequence quality scores (overall, missing data, mixed sites, private mutations, SNP clusters, frameshifts, stop codons scores, and coverage). CoV-Spectrum has a comparative analysis option, allowing the dashboard to enable comparisons of data for different variants or multiple variants against a baseline [45]. This is particularly useful for mutation dashboards, as the volume of information presented can be overwhelming. This provides users with the option to explore specific mutation data or to control which variants are compared [45].

## 5. Technologies behind Genomic Epidemiology Dashboards

### 5.1. Computational Architecture

The programming language used to develop each type of genomic epidemiology dashboard is variable and is usually based on the discretion of the developers (Table 1). There is no standard for the type of programming language used, but it is rather dependent on what the dashboard aims to achieve. There can also be variability in the programming languages used between the user interface (front-end) and the processing layer (back-end) of the dashboard. This allows developers to utilize different libraries on each end to achieve the desired result (Table 1). Panel 3 in Figure 1 presents examples of libraries and software that can be used to create the visualizations on the dashboard. Panel 4 in Figure 1 displays the various options of developers when creating the front- and back-end of genomic epidemiology dashboards. An example of this can be seen in Cluster-Tracker [39]. The back-end of the dashboard is coded in Python, whereas the front end is coded in Vanilla JavaScript, which can be described as plain JavaScript that does not contain external frameworks or libraries [39,46]. The interactive visualization on the dashboard uses the JavaScript library Leaflet. The interactive table found beneath the map visualization is enabled through DataTables, which is a plug-in for the JavaScript library, jQuery. The input files required to run these two interactive analyses are a phylogenetic tree (a Newick file), sample identification labels, and geographic information in a GeoJSON file [39]. The SARS-CoV-2 Africa dashboard is another relevant example that utilizes Python for the front- and back-end. In this dashboard, the web interface and its components were coded using Streamlit, which is a Python-based framework for web applications. The back-end is a combination of pure Python and Pandas, used to process and format data coming from GISAID. The visualizations on the dashboard were generated using Plotly [36].

Genomic epidemiology dashboards can also be supported by custom software. An example of this is Nextstrain’s dashboard, which is maintained by the ncov workflow. This workflow utilizes Augur subcommands, required for the bioinformatic pipeline, and Auspice to produce the dashboard [36]. The ncov workflow is managed by Snakemake workflow management software [47]. Augur is a bioinformatics toolkit. It allows for the phylogenetic analysis of human pathogens, as various subcommands are packaged into one command line interface tool [48]. Certain Augur subcommands have integrated pre-existing bioinformatics tools such as FastTree [49], RAxML [50], MAFFT [51], TreeTime [52], and IQTREE [53]. Finally, Auspice is used to view the annotated phylogenies in a Graphical User Interface (GUI) such as a Nextstrain dashboard [48]. The ncov workflow is open-source, allowing users such as bioinformaticians to provide it with their dataset and view it in a Nextstrain dashboard. Thus, phylogenetic analyses can be carried out with greater ease, as all the bioinformatics tools (pipeline and visualization) are encompassed in one workflow. Augur and Auspice make it possible for any bioinformatician to create a phylogenetic workflow for any pathogen, analyze the data, and visualize them in a dashboard.

### 5.2. Data Storage and Management

Genomic epidemiology dashboards can employ various ways to store and manage the data that are presented. The data can be retrieved from various storage and management systems through Application Programming Interfaces (API), which allow the dashboard to be updated regularly.

Firstly, the simplest way to store and provide data to a dashboard is through traditional file systems. The retrieved and processed data can be converted into data files (such as Excel, comma-separated values, text, and JSON) and then stored in local directories. Thus, dashboard applications will be able to read the data files by using native or third-party libraries. The SARS-CoV-2 Africa dashboard is an example that employs this system. The dashboard then utilizes an API agreement with GISAID that allows for data retrieval every day [36].

Secondly, data can be stored and managed using databases. Integrating a database system into a genomic epidemiology dashboard is effective for storing and managing large amounts of data. This prevents the dashboard from using files to store, read, process, and write data [54]. There are various types of databases used by genomic epidemiology dashboards (Table 1). The first type of database employed is the traditional Relational Database Management System (RDBMS). For example, CoV-Spectrum utilizes a PostgreSQL database [20]. This database is open-source and does not require a license. This type of database follows a server/client model, which allows for a lighter client library and ensures that clients are not affected by changes in the database engine [55]. Additionally, to retrieve information from the PostgreSQL database, the CoV-Spectrum dashboard uses two REST Application Programming Interfaces (API) for sequence and non-sequence data retrieval. The sequence data are queried using the Lightweight API for Sequences (LAPISs). The non-sequence data are retrieved from a server application managed by CoV-Spectrum. These servers are coded using the Spring Boot framework in Java and Kotlin [20].

The second type of database used in genomic epidemiology dashboards includes a non-relational database. For example, Microreact employs a MongoDB database to store the dashboard’s data [37]. A MongoDB database includes the use of documents when storing data; these documents are comparable to JavaScript Object Notation (JSON) objects [56]. MongoDB is a non-relational database that offers advantages over traditional relational databases. These include the ability to store data with no schema enforcement, which allows it to be more flexible and mirror different data types, ensuring better performance overall [56]. Relational and non-relational database records can be used in a variety of programming languages. However, the flexibility of the non-relational database makes it better suited for use cases where the data structure is dynamic and may be represented in different ways.

### 5.3. Open-Source and Adaptability

Software can be defined as open-source if its source code is available. This enables users and developers to produce their dashboards by adapting, customizing, or improving the original code. Nextstrain (https://github.com/nextstrain/augur, accessed on 10 March 2024; https://github.com/nextstrain/auspice, accessed on 10 March 2024), Cluster-Tracker (https://github.com/jmcbroome/introduction-website, accessed on 12 March 2024), and the CoV-Spectrum (https://github.com/GenSpectrum/cov-spectrum-website, accessed on 12 March 2024) dashboards are examples of dashboards that are fully open-source [20,39,44]. The SARS-CoV-2 Africa dashboard is an example of a dashboard that became an open-source software by releasing the code as the Genomic Dash framework. This is useful as users can download the Genomic Dash GitHub repository (https://github.com/BIA-lab/genomic-dash, accessed on 12 March 2024), use their pathogen data in the set format, and create their pathogen dashboard with the same analyses and visualizations as the SARS-CoV-2 Africa dashboard [36].

Genomic epidemiology dashboards can also be closed source, which prevents public access to their source code. This could be attributed to various concerns, such as privacy issues regarding sensitive information. Another concern includes the ownership of genomic data when heterogeneous data sources are utilized to curate the dashboard dataset [57]. Microreact is an example of a dashboard that is not open source but allows users to upload their pathogen data to create a replica of the Microreact dashboard. This option would be useful for users with limited computational skills who need to visualize data [37]. A drawback of this option prevents users from customizing the analyses displayed on the dashboard.

## 6. Discussion

SARS-CoV-2 genomic epidemiology dashboards are diverse, but they can be broadly classified into the three broad categories of phylogenetic, genomic surveillance, and mutational dashboards. Understanding the information presented on each dashboard category allows users to make informed decisions as to which dashboard category to use to obtain the desired information. Phylogenetic dashboards would be used by genomic specialists, such as medical virologists or epidemiologists who want to understand the evolutionary history and transmission dynamics of the virus. Genomic surveillance dashboards are designed for users ranging from laymen (public, public health officials, stakeholders, local or national ministers of health) who want to view a summary of the landscape of the epidemic or outbreak in visual form. This type of dashboard can also be used by genomic specialists who want a spatiotemporal overview of the SARS-CoV-2 variants. Lastly, mutational dashboards can be utilized by molecular biologists or virologists who can understand the large amount of mutational variation on each variant. Other users include laboratory staff, bioinformaticians, geneticists, pathologists, clinicians, infectious disease specialists, etc.

The genomic epidemiology dashboards evaluated in this review utilize reliable data sources and present diverse genomic information. The data found on genomic epidemiology dashboards are sourced from the three most used genomic databases—GenBank, GISAID, and COG-UK [58]. Genomic epidemiology dashboards in the various categories present different KPIs, analyses, and filtering options. These attributes may be similar in dashboards of the same category. Users benefit from understanding these attributes in each category so that an informed decision can be made about which would be the most useful in a given scenario. Other users may want to develop their dashboards to present different pathogen data. Therefore, it would be beneficial for the user to understand the computational architecture, data storage options, and adaptability of current genomic epidemiology dashboards. These genomic epidemiology dashboards can provide information to a variety of audiences, ranging from laymen to scientific specialists. This diversity of information provides a well-rounded understanding of the genomic epidemiology of the virus. 

Genomic epidemiology is a powerful resource that can be used to inform, understand, and respond to future outbreaks and epidemics [59,60]. The conclusions drawn from these analyses enable the early detection of emerging pathogenic variants, elucidate the spatiotemporal patterns of pathogenic variants, inform genomic surveillance programs, and initiate epidemiological investigations [61,62]. As genomic epidemiology is continually used to track outbreaks and epidemics, the success of the surveillance programs and analyses can be directly linked to the timeliness in which the data are released [15]. Thus, dashboards that characterize the genomic epidemiology of ongoing, emerging, and re-emerging pathogens are useful tools that can influence public health decisions, aiding in the response to future outbreaks and epidemics.

An important strength of genomic epidemiology dashboards lies in their adaptability to present information on novel, emerging, or re-emerging pathogens. Nextstrain and Microreact are the best examples of genomic epidemiology dashboards that have adapted their computational architecture to communicate surveillance data of different pathogens in near-real time. Nextstrain was produced to serve as a model for sharing public data. It initially shared phylogenetic analyses of dengue, seasonal and avian influenza, Zika, and Ebola [63]. The Nextstrain team was then able to quickly pivot to novel and emerging viruses, such as SARS-CoV-2 and Monkeypox, due to their robust computational architecture. Nextstrain has also created an option for users to create dashboards with their genomic surveillance data. There is extensive documentation provided by the Nextstrain team that enables users to create their own pathogen bioinformatic workflows, resulting in the production of a genomic epidemiology dashboard [63].

Microreact was a tool produced for researchers to share open genomic epidemiology data visualizations. This enables public sequencing projects of pathogens to be worked on in a collaborative and interdisciplinary effort [37]. This framework and tool were well established before the SARS-CoV-2 pandemic began, so Microreact was able to swiftly and successfully adapt it to present SARS-CoV-2 genomic data [64]. In addition, there are currently 11 published dataset dashboards that are openly available on the Microreact web application, such as the West African Ebola epidemic [65], Zika virus in the Americas [66], and *V. cholera* [67].

Genomic epidemiology dashboards can be used to respond to future outbreaks and epidemics by presenting data in near-real time. For example, the dengue virus continues to cause ongoing epidemics globally [68]. The London School of Hygiene and Tropical Medicine is currently working on the first real-time database to communicate dengue outbreak forecasting information [69]. Another pathogen that caused rapid global spread in 2022 was the mpox virus [70]. Nextstrain was successful in producing a genomic epidemiology dashboard to monitor the ongoing outbreaks (https://nextstrain.org/mpox/clade-IIb, accessed on 30 March 2024) [63].

The usefulness of genomic epidemiology dashboards is apparent; however, some limitations exist (Table 1). Firstly, dashboards are rendered irrelevant if they are not updated and maintained. For example, the Microreact and COVID-19 Genomic Surveillance dashboard by Wellcome Sanger Institute was last updated in February 2022 and February 2023, respectively (Table 1). Thus, the usefulness of genomic epidemiology dashboards can be short-lived and only relevant during an outbreak or epidemic. Secondly, dashboards do not allow for the data to be downloaded if they are sourced from GISAID. This prevents users from accessing and interacting with the data being presented. It is important to acknowledge that this review did not employ a systematic approach to select the dashboards reviewed. Not all SARS-CoV-2 genomic dashboards were included, thus it cannot be interpreted as exhaustive. A study by Cheng et al. 2023 explores the different web resources for genomic database, annotation, analysis, and variant tracking for SARS-CoV-2 and provides more examples of genomic dashboards [58].

## 7. Conclusions

Genomic epidemiology dashboards present intricate genomic surveillance data and analyses in digestible visualizations, which ensures this information is communicated effectively. There are three main types of genomic epidemiology dashboards that source data from various databases, present different analyses through easy-to-interpret visualizations, and employ robust computational architecture. Genomic epidemiology dashboards have the potential to empower stakeholders, policy decision-makers, and public health officials to make decisions based on the genomics of an underlying outbreak or epidemic. The future of genomic epidemiology dashboards is bright, given that they are produced in near-real time for novel, emerging, and re-emerging pathogens.

## Figures and Tables

**Table 1:** Summary of the data, visual features, functional features, and technologies behind six SARS-CoV-2 genomic epidemiology dashboards.

Dashboard Attributes	Nextstrain’s Genomic Epidemiology of SARS-CoV-2 Dashboard ^1^	Cluster-Tracker ^2^	Global Distribution of SARS-CoV-2 Microreact Dashboard ^3^	COVID-19 Genomic Surveillance Dashboard ^6^	SARS-CoV-2 Africa Dashboard ^8^	CoV-Spectrum ^9^
**Maintained by**	Nextstrain team	Biomolecular Engineering and Genomics Institute, University of California	The Centre for Genomic Pathogen Surveillance	Wellcome Sanger Institute	Centre for Epidemic Response and Innovation	Computational Evolution group at ETH Zurich
**Main purpose**	Presents the genomic epidemiology of SARS-CoV-2 using phylogenetic analyses	Communicates the results of a phylogenetically informed summary heuristic for the USA	Shares the genomic epidemiology of SARS-CoV-2 and enables open data visualization in a collaborative and interdisciplinary effort	Continually evaluates and understands the state of the pandemic and the circulating lineages of SARS-CoV-2 in England	Produces an interactive perspective of the SARS-CoV-2 variants circulating on the African continent	Identifies novel VOCs and tracks the spread of known VOCs
**Type**	Phylogenetic	Phylogenetic	Genomic surveillance	Genomic surveillance	Genomic surveillance	Mutational
**Update frequency**	Every two days	ND	Last updated: 11 February 2022	Last updated: 20 February 2023	Every day	Once a week
**Open-source**	Yes	Yes	No	Uses open-source framework—CovInce ^7^	Uses open-source framework—Genomic Dash	Yes
**Data source**	GenBank and GISAID	GenBank, GISAID, and COG-UK	COG-UK	COG-UK	GISAID	GISAID and Nextstrain open data
**Data availability**	GenBank data only	Yes	Yes	Yes	No	Yes
**Geographic level**	Global	National—USA	Global	National—England	Regional—Africa	Global
**Key performance indicators**	Phylogenetic tree, proportion of variants, nucleotide diversity	Number of clusters and introductions	Number of genomes, proportion of variants, number of cases	Proportion of lineages, estimated cases, number of genomes	Number of genomes, proportion of lineages, proportion of lineages per country over time	Total sequences, overall proportion, descendent lineages, estimated cases, relative growth advantage, reproduction number, age demographics, mutations over time, nucleotide entropy, insertions, substitutions, and deletions
**Analyses**	Phylogenetic and mutational	Phylogenetically informed summary heuristic	Genomic pattern	Genomic pattern	Genomic pattern	Mutational
**Filtering options**	Dataset, time, variant, country, clade, emerging lineage, genotype, etc.	Time	Time and variant	Time, local authorities in England, and lineages	Time, region, country, and lineages	Dataset, time, host, submission dates, and sequence quality
**Computational architecture**	Front-end: Aupice Back-end: Augur	Front-end: Vanilla JavaScript Back-end: Python Figures: Javascript library—Leaflet and jQuery (DataTables)	Front-end: React and Node.js frameworks Back-end: JavaScript, CSS ^4^ and HTML ^5^ Figures: JavaScript libraries—Phylocanvas, vis.js, Leaflet, and Mapbox mapping platform	Front-end: React framework Back-end: CovInce framework	Front-end: Streamlit Figures: Python library—Plotly	Front-end: React framework Back-end: TypeScript language Figures: Rechart and Python library—Plotly
**Databases/API**	ND	None	MongoDB	ND	GISAID API	PostgreSQL and REST APIs
**Limitations**	The pie charts are difficult to read as there are a lot of segments to display for the different variants	This summary heuristic cannot be filtered for by variant	The data presented on the global map are not standardized, which leads to most countries appearing to have produced minimal or no sequences	The dashboard has not been maintained and updated	It has a slower loading time once filters are applied	There are no figures to consolidate the temporal or geographical information of the sequences

^1^ https://nextstrain.org/ncov/gisaid/global/6m, accessed on 1 March 2024, ^2^ https://clustertracker.gi.ucsc.edu, accessed on 1 March 2024, ^3^ https://microreact.org/project/6Y5GvktuhqgRRDwRQ7cUze-global-sars-cov-2-2019-12-242022-02-11, accessed on 1 March 2024, ^4^ Cascading Style Sheets, ^5^ HyperText Markup Language, ^6^ https://covid19.sanger.ac.uk/lineages/raw, accessed on 1 March 2024, ^7^ https://github.com/covince/covince?tab=readme-ov-file, accessed on 20 March 2024, ^8^ https://covid-africa.genomic-dash.app/, accessed on 1 March 2024, ^9^ https://cov-spectrum.org/explore/World/AllSamples/Past6M, accessed on 1 March 2024.

## Data Availability

No new data were created or analyzed in this study. Data sharing is not applicable to this review.

## Supplementary Materials

The following supporting information can be downloaded at: https://www.mdpi.com/article/10.3390/genes15070876/s1, Figure S1: Nextstrain SARS-CoV-2 Global dashboard depicts the genomic epidemiology of the SARS-CoV-2 pandemic; Figure S2: Cluster-Tracker dashboard; Figure S3: SARS-CoV-2 Africa dashboard presenting genomic SARS-CoV-2 information for the African continent; Figure S4: Main page on CoV-Spectrum dashboard; Figure S5: Wellcome Sanger Institute COVID-19 Genomic Surveillance dashboard; Figure S6: CoV-Spectrum dashboard advanced filters.

## References

[1] Holmes B.J. (2008). Communicating about emerging infectious disease: The importance of research. Health Risk Soc..

[2] M’ikanatha N.M., Lynfield R., Julian K.G., Van Beneden C.A., Valk H.d. (2013). Infectious disease surveillance: A cornerstone for prevention and control. Infectious Disease Surveillance.

[3] Friede A., Blum H.L., McDonald M. (1995). Public health informatics: How information-age technology can strengthen public health. Annu. Rev. Public Health.

[4] Thacker S.B., Stroup D.F. (2013). Origins and progress in surveillance systems. Infectious Disease Surveillance.

[5] Walker D.M., Yeager V.A., Lawrence J., McAlearney A.S. (2021). Identifying Opportunities to Strengthen the Public Health Informatics Infrastructure: Exploring Hospitals’ Challenges with Data Exchange. Milbank Q..

[6] Aziz H.A. (2017). A review of the role of public health informatics in healthcare. J. Taibah Univ. Med. Sci..

[7] Matheus R., Janssen M., Maheshwari D. (2020). Data science empowering the public: Data-driven dashboards for transparent and accountable decision-making in smart cities. Gov. Inf. Q..

[8] Ivanković D., Barbazza E., Bos V., Brito Fernandes Ó., Jamieson Gilmore K., Jansen T., Kara P., Larrain N., Lu S., Meza-Torres B. (2021). Features Constituting Actionable COVID-19 Dashboards: Descriptive Assessment and Expert Appraisal of 158 Public Web-Based COVID-19 Dashboards. J. Med. Internet Res..

[9] Vahedi A., Moghaddasi H., Asadi F., Hosseini A.S., Nazemi E. (2022). Applications, features and key indicators for the development of COVID-19 dashboards: A systematic review study. Inform. Med. Unlocked.

[10] Deng X., Gu W., Federman S., du Plessis L., Pybus O.G., Faria N.R., Wang C., Yu G., Bushnell B., Pan C.-Y. (2020). Genomic surveillance reveals multiple introductions of SARS-CoV-2 into Northern California. Science.

[11] Tegally H., Wilkinson E., Giovanetti M., Iranzadeh A., Fonseca V., Giandhari J., Doolabh D., Pillay S., San E.J., Msomi N. (2021). Detection of a SARS-CoV-2 variant of concern in South Africa. Nature.

[12] Wilkinson E., Giovanetti M., Tegally H., San J.E., Lessells R., Cuadros D., Martin D.P., Rasmussen D.A., Zekri A.-R.N., Sangare A.K. (2021). A year of genomic surveillance reveals how the SARS-CoV-2 pandemic unfolded in Africa. Science.

[13] Dong E., Du H., Gardner L. (2020). An interactive web-based dashboard to track COVID-19 in real time. Lancet Infect. Dis..

[14] World Health Organization WHO COVID-19 Dashboard. https://data.who.int/dashboards/covid19/cases?n=c.

[15] Lo S.W., Jamrozy D. (2020). Genomics and epidemiological surveillance. Nat. Rev. Microbiol..

[16] Tegally H., Moir M., Everatt J., Giovanetti M., Scheepers C., Wilkinson E., Subramoney K., Makatini Z., Moyo S., Amoako D.G. (2022). Emergence of SARS-CoV-2 Omicron lineages BA.4 and BA.5 in South Africa. Nat. Med..

[17] GISAID. https://gisaid.org/.

[18] Inzaule S.C., Tessema S.K., Kebede Y., Ogwell Ouma A.E., Nkengasong J.N. (2021). Genomic-informed pathogen surveillance in Africa: Opportunities and challenges. Lancet Infect. Dis..

[19] Gardy J.L., Loman N.J. (2018). Towards a genomics-informed, real-time, global pathogen surveillance system. Nat. Rev. Genet..

[20] Chen C., Nadeau S., Yared M., Voinov P., Xie N., Roemer C., Stadler T. (2021). CoV-Spectrum: Analysis of globally shared SARS-CoV-2 data to identify and characterize new variants. Bioinformatics.

[21] Kalinich C.C., Jensen C.G., Neugebauer P., Petrone M.E., Peña-Hernández M., Ott I.M., Wyllie A.L., Alpert T., Vogels C.B.F., Fauver J.R. (2020). Real-time public health communication of local SARS-CoV-2 genomic epidemiology. PLoS Biol..

[22] Fareed N., Swoboda C.M., Chen S., Potter E., Wu D.T.Y., Sieck C.J. (2021). U.S. COVID-19 State Government Public Dashboards: An Expert Review. Appl. Clin. Inform..

[23] Dixit R.A., Hurst S., Adams K.T., Boxley C., Lysen-Hendershot K., Bennett S.S., Booker E., Ratwani R.M. (2020). Rapid development of visualization dashboards to enhance situation awareness of COVID-19 telehealth initiatives at a multihospital healthcare system. J. Am. Med. Inform. Assoc..

[24] Mathieu E., Ritchie H., Rodés-Guirao L., Appel C., Giattino C., Hasell J., Macdonald B., Dattani S., Beltekian D., Ortiz-Ospina E. Coronavirus Pandemic (COVID-19). https://ourworldindata.org/coronavirus.

[25] Parolini N., Ardenghi G., Dede’ L., Quarteroni A. (2021). A mathematical dashboard for the analysis of Italian COVID-19 epidemic data. Int. J. Numer. Methods Biomed. Eng..

[26] Stevens J.S., Toma K., Tanzi-Pfeifer S., Rao M.K., Mohan S., Gharavi A.G., Radhakrishnan J. (2020). Dashboards to Facilitate Nephrology Disaster Planning in the COVID-19 Era. Kidney Int. Rep..

[27] Attwood S.W., Hill S.C., Aanensen D.M., Connor T.R., Pybus O.G. (2022). Phylogenetic and phylodynamic approaches to understanding and combating the early SARS-CoV-2 pandemic. Nat. Rev. Genet..

[28] Chen Z., Azman A.S., Chen X., Zou J., Tian Y., Sun R., Xu X., Wu Y., Lu W., Ge S. (2022). Global landscape of SARS-CoV-2 genomic surveillance and data sharing. Nat. Genet..

[29] Wang R., Chen J., Gao K., Hozumi Y., Yin C., Wei G.W. (2021). Analysis of SARS-CoV-2 mutations in the United States suggests presence of four substrains and novel variants. Commun. Biol..

[30] Makowski E.K., Schardt J.S., Smith M.D., Tessier P.M. (2022). Mutational analysis of SARS-CoV-2 variants of concern reveals key tradeoffs between receptor affinity and antibody escape. PLoS Comput. Biol..

[31] Nijkamp P., Kourtit K. (2022). Place-Specific Corona Dashboards for Health Policy: Design and Application of a ‘Dutchboard’. Sustainability.

[32] Clark K., Karsch-Mizrachi I., Lipman D.J., Ostell J., Sayers E.W. (2016). GenBank. Nucleic Acids Res..

[33] Shu Y., McCauley J. (2017). GISAID: Global initiative on sharing all influenza data-from vision to reality. Euro Surveill..

[34] Bedford T., Hadfield J., Hodcroft E., Huddleston J., Neher R., Sibley T. (2021). Extension of SARS-CoV-2 Data Processing to Incorporate Open Data through GenBank. https://dev.nextstrain.org/blog/2021-07-08-ncov-open-announcement.

[35] Blaxter M., Danchin A., Savakis B., Fukami-Kobayashi K., Kurokawa K., Sugano S., Roberts R.J., Salzberg S.L., Wu C.I. (2016). Reminder to deposit DNA sequences. Science.

[36] Xavier J.S., Moir M., Tegally H., Sitharam N., Abdool Karim W., San J.E., Linhares J., Wilkinson E., Ascher D.B., Baxter C. (2023). SARS-CoV-2 Africa dashboard for real-time COVID-19 information. Nat. Microbiol..

[37] Argimón S., Abudahab K., Goater R.J.E., Fedosejev A., Bhai J., Glasner C., Feil E.J., Holden M.T.G., Yeats C.A., Grundmann H. (2016). Microreact: Visualizing and sharing data for genomic epidemiology and phylogeography. Microb. Genom..

[38] Wellcome Sanger Institute COVID-19 Genomic Surveillance. https://covid19.sanger.ac.uk/lineages/raw.

[39] McBroome J., Martin J., de Bernardi Schneider A., Turakhia Y., Corbett-Detig R. (2022). Identifying SARS-CoV-2 regional introductions and transmission clusters in real time. Virus Evol..

[40] Lechner B., Fruhling A. (2014). Towards public health dashboard design guidelines. Proceedings of the Lecture Notes in Computer Science.

[41] Cahyadi A., Prananto A. (2015). Reflecting design thinking: A case study of the process of designing dashboards. J. Syst. Inf. Technol..

[42] Maury E., Boldi M.-O., Greub G., Chavez V., Jaton K., Opota O. (2021). An Automated Dashboard to Improve Laboratory COVID-19 Diagnostics Management. Front. Digit. Health.

[43] Mattern S. (2015). Mission Control: A History of the Urban Dashboard. https://placesjournal.org/article/mission-control-a-history-of-the-urban-dashboard/.

[44] Nextstrain Team Genomic Epidemiology of SARS-CoV-2 with Subsampling Focused Globally over the Past 6 Months. https://nextstrain.org/ncov/gisaid/global/6m.

[45] CoV-Spectrum Detect and Analyze Variants of SARS-CoV-2. https://cov-spectrum.org/explore/World/AllSamples/Past6M.

[46] Persson M. (2020). JavaScript DOM Manipulation Performance: Comparing Vanilla JavaScript and Leading JavaScript Front-End Frameworks. Diploma Thesis.

[47] Köster J., Rahmann S. (2012). Snakemake—A scalable bioinformatics workflow engine. Bioinformatics.

[48] Huddleston J., Hadfield J., Sibley T.R., Lee J., Fay K., Ilcisin M., Harkins E., Bedford T., Neher R.A., Hodcroft E.B. (2021). Augur: A bioinformatics toolkit for phylogenetic analyses of human pathogens. J. Open Source Softw..

[49] Price M.N., Dehal P.S., Arkin A.P. (2010). FastTree 2—Approximately Maximum-Likelihood Trees for Large Alignments. PLoS ONE.

[50] Stamatakis A. (2014). RAxML version 8: A tool for phylogenetic analysis and post-analysis of large phylogenies. Bioinformatics.

[51] Katoh K., Misawa K., Kuma K., Miyata T. (2002). MAFFT: A novel method for rapid multiple sequence alignment based on fast Fourier transform. Nucleic Acids Res..

[52] Sagulenko P., Puller V., Neher R.A. (2018). TreeTime: Maximum-likelihood phylodynamic analysis. Virus Evol..

[53] Nguyen L.T., Schmidt H.A., von Haeseler A., Minh B.Q. (2015). IQ-TREE: A fast and effective stochastic algorithm for estimating maximum-likelihood phylogenies. Mol. Biol. Evol..

[54] Batini C., Lenzerini M., Navathe S. (1986). A Comparative Analysis of Methodologies for Database Schema Integration. ACM Comput. Surv..

[55] Jung M.G., Youn S.A., Bae J., Choi Y.L. A Study on Data Input and Output Performance Comparison of MongoDB and PostgreSQL in the Big Data Environment. Proceedings of the 2015 8th International Conference on Database Theory and Application (DTA).

[56] Győrödi C., Gyorodi R., Pecherle G., Olah A. A Comparative Study: MongoDB vs. MySQL. Proceedings of the 13th International Conference on Engineering of Modern Electric Systems (EMES).

[57] Velmovitsky P.E., Bevilacqua T., Alencar P., Cowan D., Morita P.P. (2021). Convergence of Precision Medicine and Public Health Into Precision Public Health: Toward a Big Data Perspective. Front. Public Health.

[58] Cheng Y., Ji C., Zhou H.-Y., Zheng H., Wu A. (2023). Web Resources for SARS-CoV-2 Genomic Database, Annotation, Analysis and Variant Tracking. Viruses.

[59] Hill V., Githinji G., Vogels C.B.F., Bento A.I., Chaguza C., Carrington C.V.F., Grubaugh N.D. (2023). Toward a global virus genomic surveillance network. Cell Host Microbe.

[60] Tang P., Croxen M.A., Hasan M.R., Hsiao W.W.L., Hoang L.M. (2017). Infection control in the new age of genomic epidemiology. Am. J. Infect. Control.

[61] Hill V., Ruis C., Bajaj S., Pybus O.G., Kraemer M.U.G. (2021). Progress and challenges in virus genomic epidemiology. Trends Parasitol..

[62] Jansz N., Faulkner G.J. (2024). Viral genome sequencing methods: Benefits and pitfalls of current approaches. Biochem. Soc. Trans..

[63] Hadfield J., Megill C., Bell S.M., Huddleston J., Potter B., Callender C., Sagulenko P., Bedford T., Neher R.A. (2018). Nextstrain: Real-time tracking of pathogen evolution. Bioinformatics.

[64] Wright D.W., Harvey W.T., Hughes J., Cox M., Peacock T.P., Colquhoun R., Jackson B., Orton R., Nielsen M., Hsu N.S. (2022). Tracking SARS-CoV-2 mutations and variants through the COG-UK-Mutation Explorer. Virus Evol..

[65] Dudas G., Carvalho L.M., Bedford T., Tatem A.J., Baele G., Faria N.R., Park D.J., Ladner J.T., Arias A., Asogun D. (2017). Virus genomes reveal factors that spread and sustained the Ebola epidemic. Nature.

[66] Faria N.R., Azevedo R., Kraemer M.U.G., Souza R., Cunha M.S., Hill S.C., Thézé J., Bonsall M.B., Bowden T.A., Rissanen I. (2016). Zika virus in the Americas: Early epidemiological and genetic findings. Science.

[67] Mutreja A., Kim D.W., Thomson N.R., Connor T.R., Lee J.H., Kariuki S., Croucher N.J., Choi S.Y., Harris S.R., Lebens M. (2011). Evidence for several waves of global transmission in the seventh cholera pandemic. Nature.

[68] Xu C., Xu J., Wang L. (2024). Long-term effects of climate factors on dengue fever over a 40-year period. BMC Public Health.

[69] AXA, UK (2023). AXA to Partner with London School of Hygiene & Tropical Medicine on Dengue Outbreak Forecasting Project. https://www.axa.co.uk/newsroom/media-releases/2023/axa-to-partner-with-london-school-of-hygiene-and-tropical-medicine-on-dengue-outbreak-forecasting-project/.

[70] Haque M.A., Halder A.S., Hossain M.J., Islam M.R. (2024). Prediction of potential public health risk of the recent multicountry monkeypox outbreak: An update after the end declaration of global public health emergency. Health Sci. Rep..

