# Supplementary material for: SARS-CoV-2 Genomic Epidemiology Dashboards: A Review of Functionality and Technological Frameworks for the Public Health Response
Source: Genes (Basel). 2024 Jul 3;15(7):876. doi: 10.3390/genes15070876 (PMC11275337; doi:10.3390/genes15070876)
Supplement: Supplementary file 1 [file genes-15-00876-s001.zip › genes-3080248-supplementary.pdf]

SARS-CoV-2 Genomic Epidemiology Dashboards: A Review of Functionality and Technological Frameworks for the Public Health Response

Nikita Sitharam [1], Houriiyah Tegally [1], Danilo de Castro Silva [1,2], Cheryl Baxter [1,3], Tulio de Oliveira [1,3,4,5,*] and Joicymara S. Xavier [1,6,7,*]

[1] Centre for Epidemic Response and Innovation (CERI), School for Data Science and Computational Thinking, Stellenbosch University, Stellenbosch 7600, South Africa; nikitasitharam1@gmail.com (N.S.)

[2] Department of Computer Science, Faculty of Science, Stellenbosch University, Stellenbosch 7600, South Africa

[3] Centre for the AIDS Programme of Research in South Africa (CAPRISA), Durban 4001, South Africa

[4] KwaZulu-Natal Research Innovation and Sequencing Platform (KRISP), Nelson R Mandela School of Medicine, University of KwaZulu-Natal, Durban 4001, South Africa

[5] Department of Global Health, University of Washington, Seattle, WA 98105, USA

[6] Institute of Agricultural Sciences, Universidade Federal dos Vales do Jequitinhonha e Mucuri (UFVJM), Unaí38610-000, Brazil

[7] Institute of Biological Sciences, Universidade Federal de Minas Gerais (UFMG), Belo Horizonte 31270-901, Brazil

* Correspondence: tulio@sun.ac.za (T.d.O.); joicy@sun.ac.za (J.S.X.)

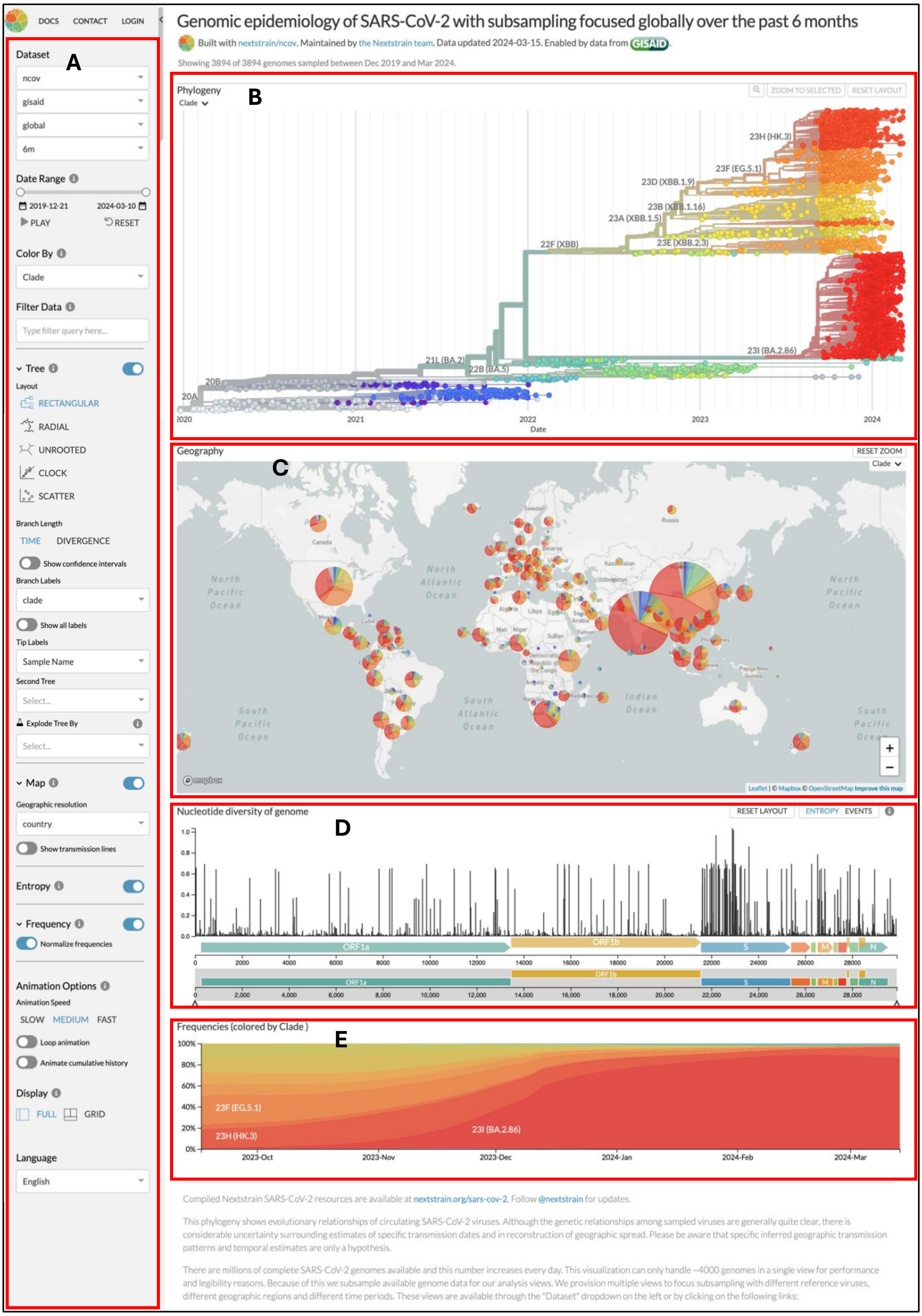

Figure S1. Nextstrain SARS-CoV-2 Global dashboard depicts the genomic epidemiology of the SARS-CoV-2 pandemic. Panel (A) – filter panel, (B) – phylogenetic tree, (C) – global map, (D) – nucleotide diversity of genome, (E) – frequency figure

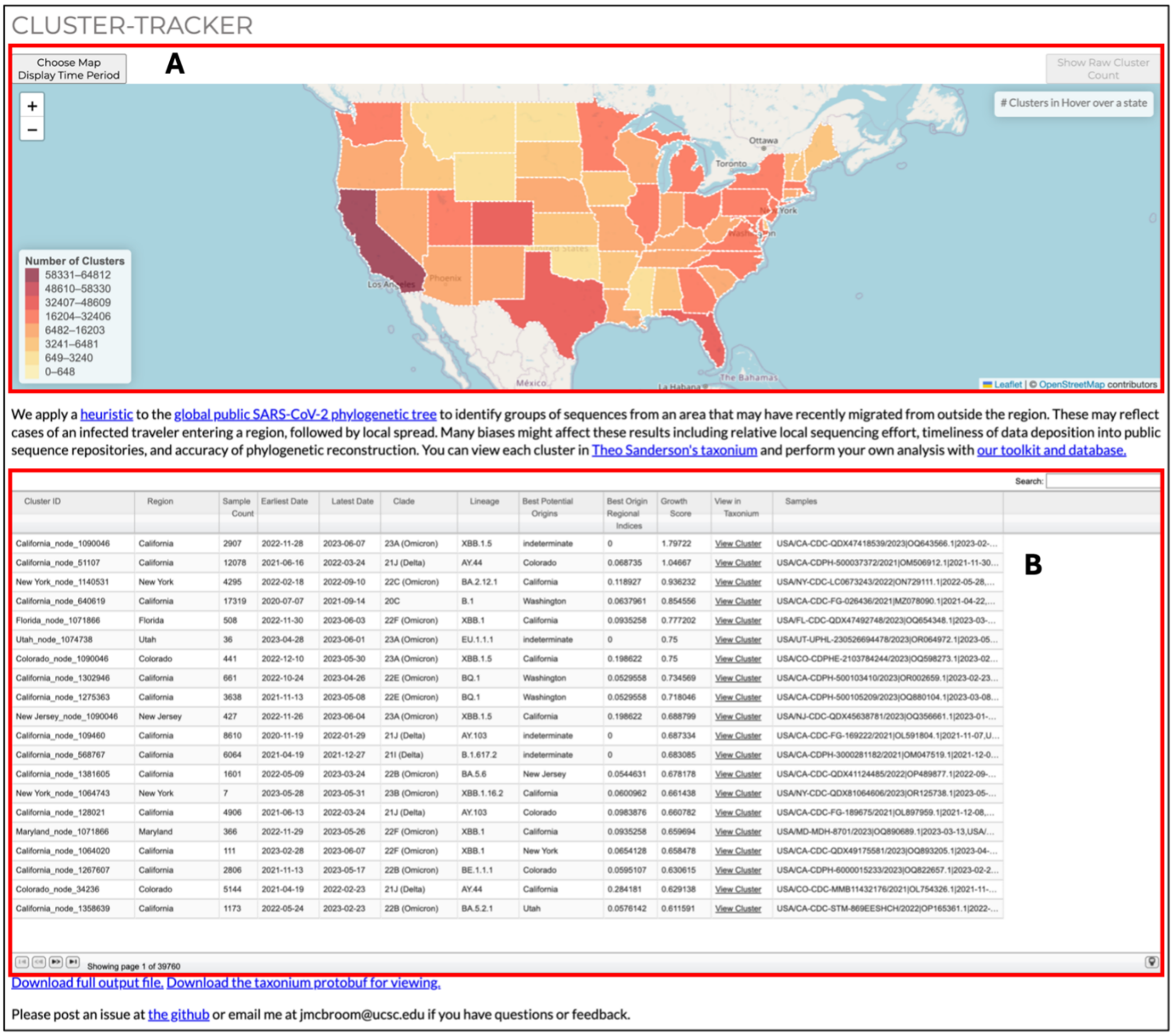

Cluster ID	Region	Sample Count	Earliest Date	Latest Date	Clade	Lineage	Best Potential Origins	Best Origin Regional Indices	Growth Score	View in Taxonium	Samples
California_node_1090046	California	2907	2022-11-28	2023-06-07	23A (Omicron)	XBB.1.5	indeterminate	0	1.79722	View Cluster	USA/CA-CDC-QDX47418539/2023|OQ643566.1|2023-02-…
California_node_51107	California	12078	2021-06-16	2022-03-24	21J (Delta)	AY.44	Colorado	0.068735	1.04667	View Cluster	USA/CA-CDPH-500037372/2021|OM506912.1|2021-11-30…
New York_node_1140531	New York	4295	2022-02-18	2022-09-10	22C (Omicron)	BA.2.12.1	California	0.118927	0.936232	View Cluster	USA/NY-CDC-LC0673243/2022|ON729111.1|2022-05-28,…
California_node_640619	California	17319	2020-07-07	2021-09-14	20C	B.1	Washington	0.0637961	0.854556	View Cluster	USA/CA-CDC-FG-026436/2021|MZ078090.1|2021-04-22,…
Florida_node_1071866	Florida	508	2022-11-30	2023-06-03	22F (Omicron)	XBB.1	California	0.0935258	0.777202	View Cluster	USA/FL-CDC-QDX47492748/2023|OQ654348.1|2023-03-…
Utah_node_1074738	Utah	36	2023-04-28	2023-06-01	23A (Omicron)	EU.1.1.1	indeterminate	0	0.75	View Cluster	USA/UT-UPHL-230526694478/2023|OR064972.1|2023-05…
Colorado_node_1090046	Colorado	441	2022-12-10	2023-05-30	23A (Omicron)	XBB.1.5	California	0.198622	0.75	View Cluster	USA/CO-CDPHE-2103784244/2023|OQ598273.1|2023-02…
California_node_1302946	California	661	2022-10-24	2023-04-26	22E (Omicron)	BQ.1	Washington	0.0529558	0.734569	View Cluster	USA/CA-CDPH-500103410/2023|OR002659.1|2023-02-23…
California_node_1275363	California	3638	2021-11-13	2023-05-08	22E (Omicron)	BQ.1	Washington	0.0529558	0.718046	View Cluster	USA/CA-CDPH-500105209/2023|OQ880104.1|2023-03-08…
New Jersey_node_1090046	New Jersey	427	2022-11-26	2023-06-04	23A (Omicron)	XBB.1.5	California	0.198622	0.688799	View Cluster	USA/NJ-CDC-QDX45638781/2023|OQ356661.1|2023-01-…
California_node_109460	California	8610	2020-11-19	2022-01-29	21J (Delta)	AY.103	indeterminate	0	0.687334	View Cluster	USA/CA-CDC-FG-169222/2021|OL591804.1|2021-11-07,U…
California_node_568767	California	6064	2021-04-19	2021-12-27	21I (Delta)	B.1.617.2	indeterminate	0	0.683085	View Cluster	USA/CA-CDPH-3000281182/2021|OM047519.1|2021-12-0…
California_node_1381605	California	1601	2022-05-09	2023-03-24	22B (Omicron)	BA.5.6	New Jersey	0.0544631	0.678178	View Cluster	USA/CA-CDC-QDX41124485/2022|OP489877.1|2022-09-…
New York_node_1064743	New York	7	2023-05-28	2023-05-31	23B (Omicron)	XBB.1.16.2	California	0.0600962	0.661438	View Cluster	USA/NY-CDC-QDX81064606/2023|OR125738.1|2023-05-…
California_node_128021	California	4906	2021-06-13	2022-03-24	21J (Delta)	AY.103	Colorado	0.0983876	0.660782	View Cluster	USA/CA-CDC-FG-189675/2021|OL897959.1|2021-12-08,…
Maryland_node_1071866	Maryland	366	2022-11-29	2023-05-26	22F (Omicron)	XBB.1	California	0.0935258	0.659694	View Cluster	USA/MD-MDH-8701/2023|OQ890689.1|2023-03-13,USA/…
California_node_1064020	California	111	2023-02-28	2023-06-07	22F (Omicron)	XBB.1	New York	0.0654128	0.658478	View Cluster	USA/CA-CDC-QDX49175581/2023|OQ893205.1|2023-04-…
California_node_1267607	California	2806	2021-11-13	2023-05-17	22B (Omicron)	BE.1.1.1	Colorado	0.0595107	0.630615	View Cluster	USA/CA-CDPH-6000015233/2023|OQ822657.1|2023-02-2…
Colorado_node_34236	Colorado	5144	2021-04-19	2022-02-23	21J (Delta)	AY.44	California	0.284181	0.629138	View Cluster	USA/CO-CDC-MMB11432176/2021|OL754326.1|2021-11-…
California_node_1358639	California	1173	2022-05-24	2023-02-23	22B (Omicron)	BA.5.2.1	Utah	0.0576142	0.611591	View Cluster	USA/CA-CDC-STM-869EESHCH/2022|OP165361.1|2022-…

Figure S2. Cluster-Tracker dashboard. Panel (A) – map of the United States of America, (B) – table containing detailed information of each cluster

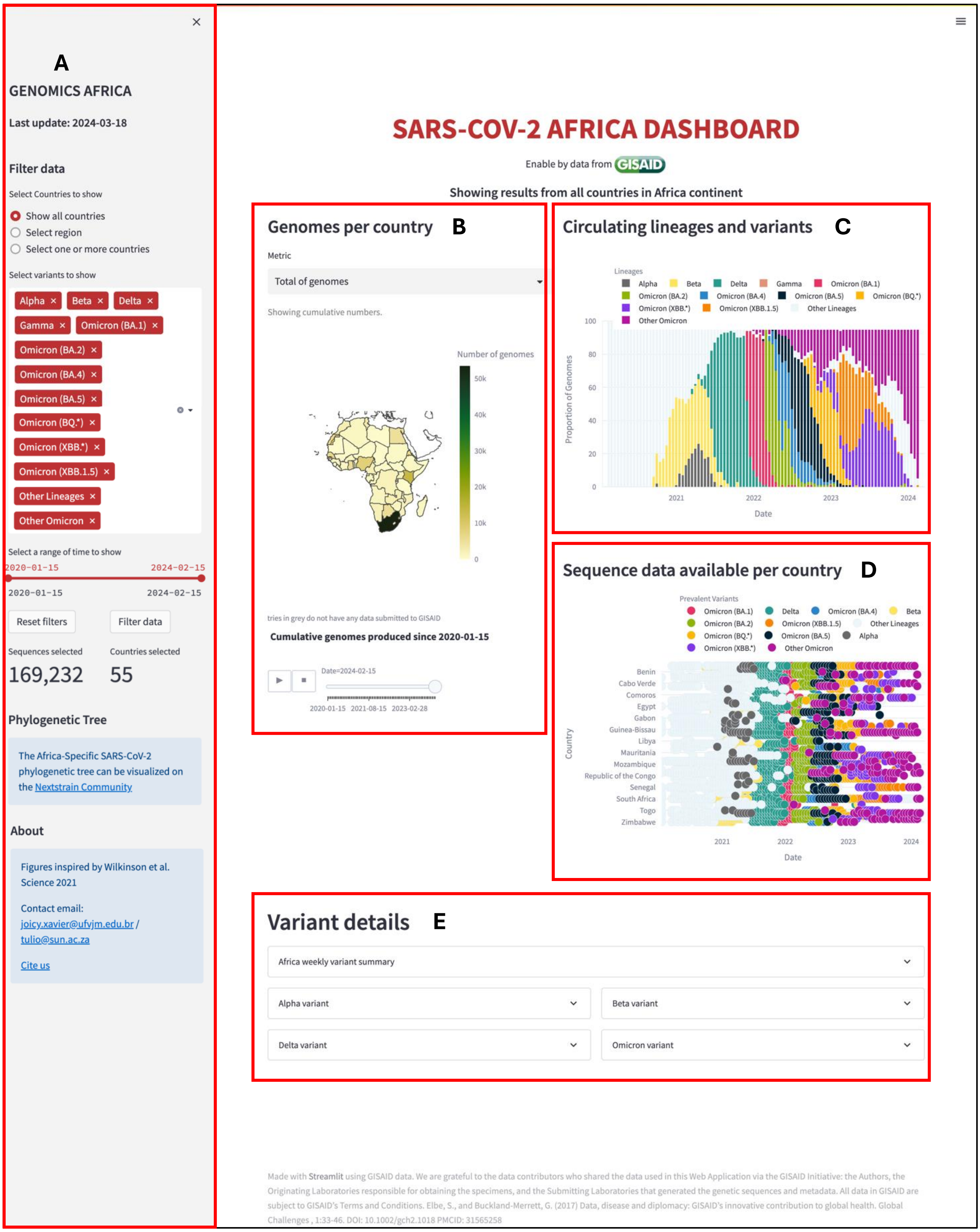

Figure S3. SARS-CoV-2 Africa dashboard presenting genomic SARS-CoV-2 information for the African continent. Panel (A) – filter sidebar, (B) – map of Africa, (C) – circulating lineages and variants, (D) – sequence data available per country, (E) – variant details menu

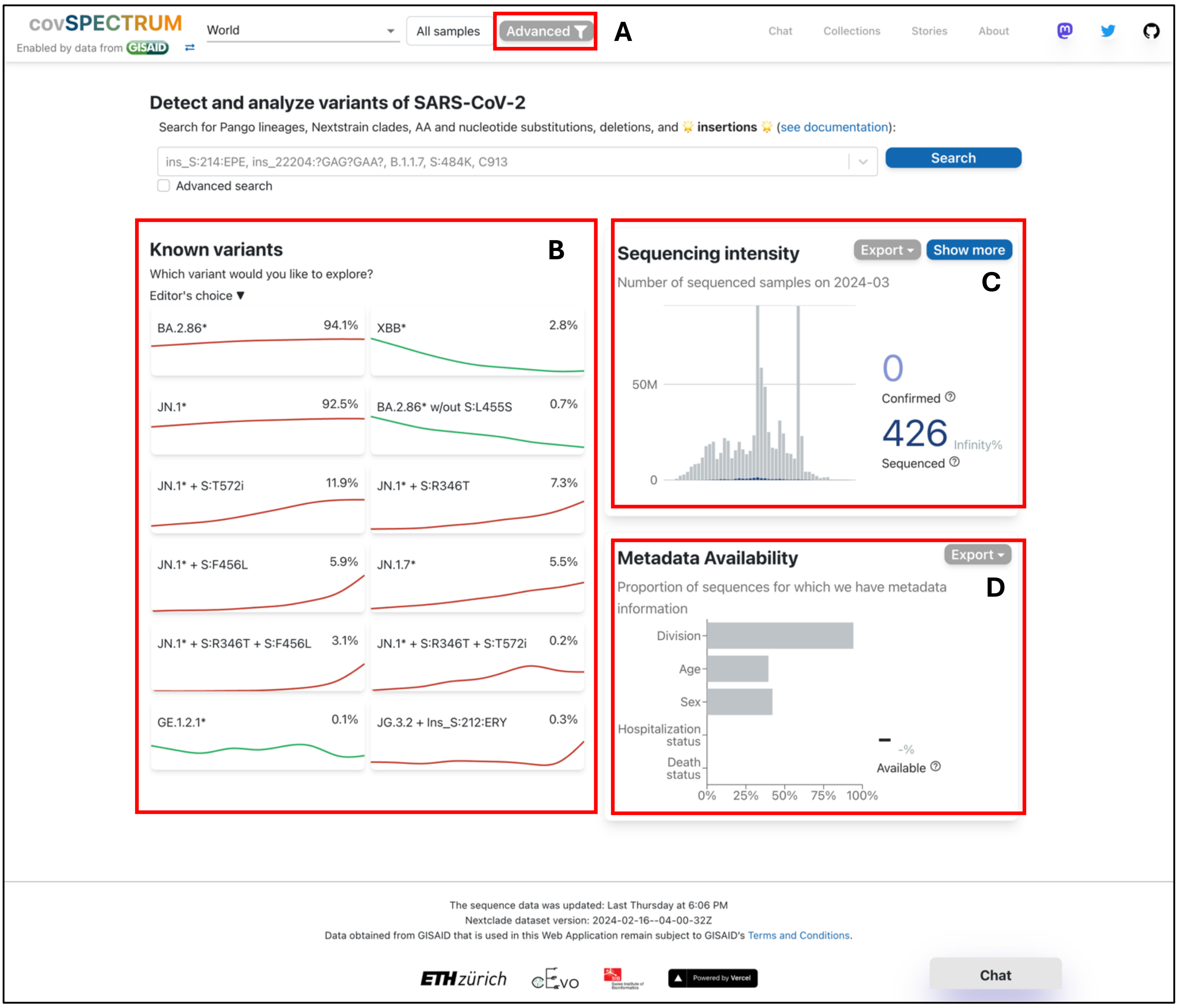

Figure S4. Main page on CoV-Spectrum dashboard. Panel (A) – advanced filter option, (B) – grid of variants, (C) – sequencing intensity figure, (D) – metadata availability figure

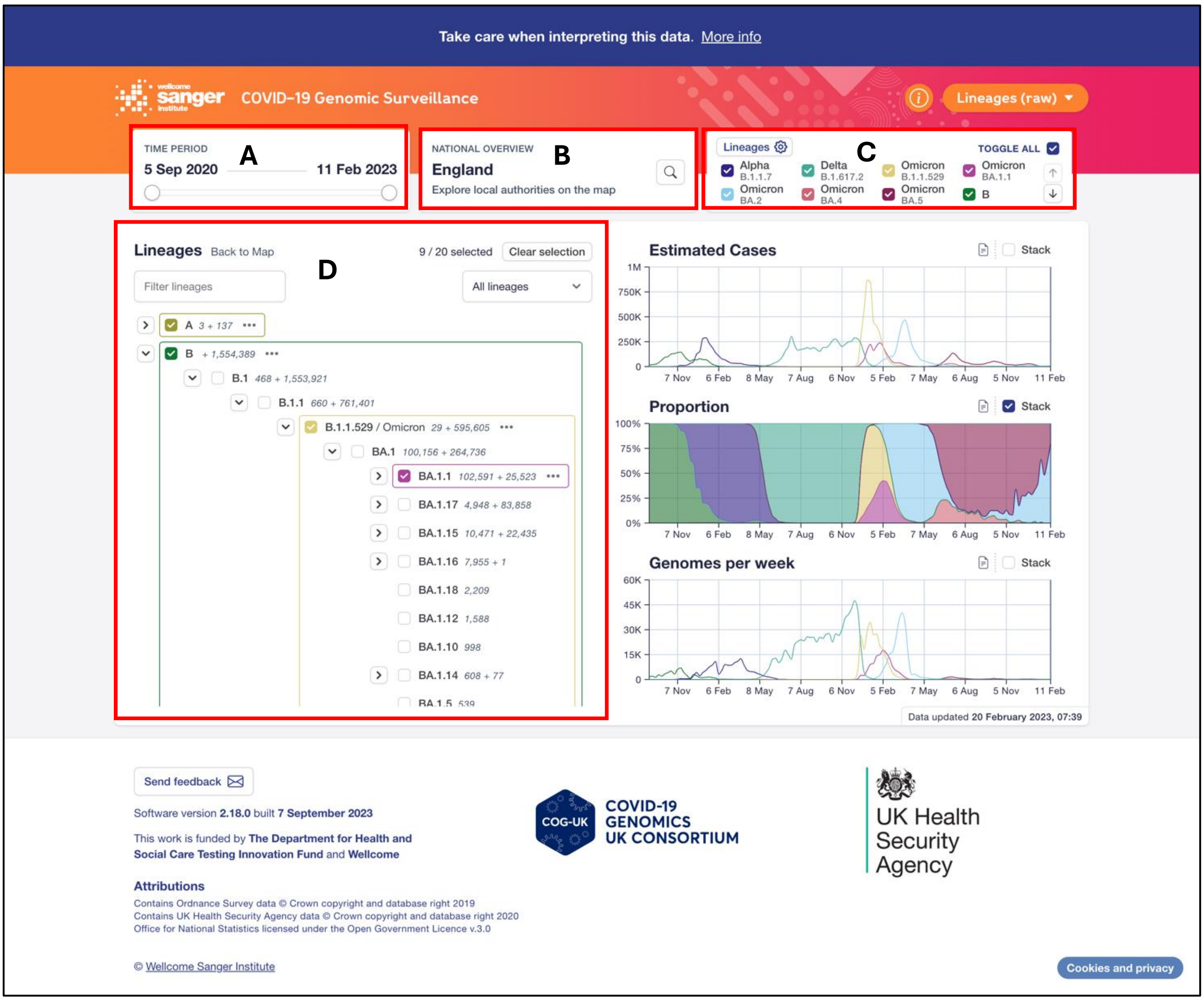

Figure S5. Wellcome Sanger Institute COVID-19 Genomic Surveillance dashboard. Panel (A) – timescale filter, (B) – local authority or postcode filter, (C) – lineages filter, (D) – lineage option map

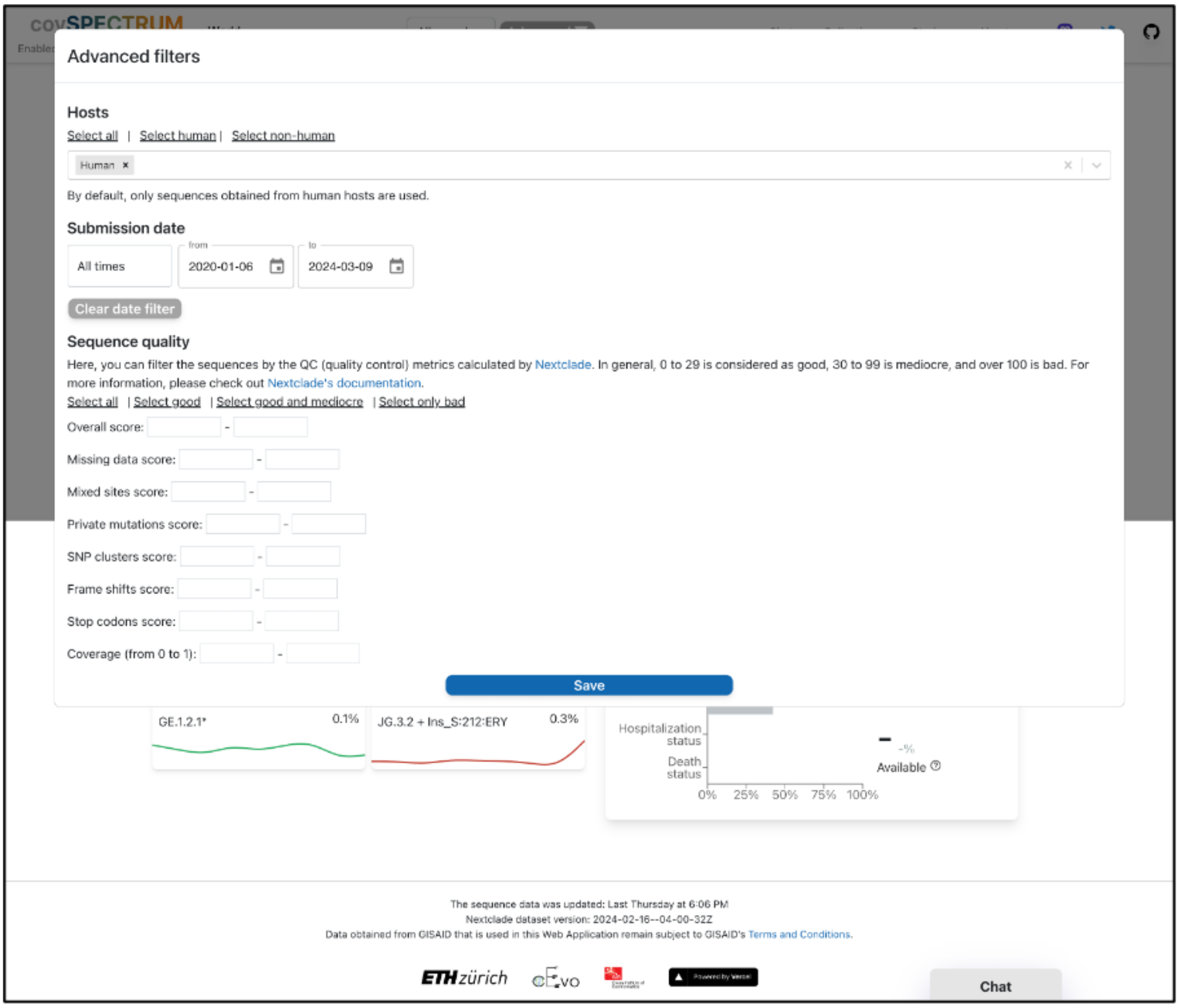

Figure S6. CoV-Spectrum dashboard advanced filters. Advanced filtering function to filter data by different hosts, submission date range, and sequence quality indicators
